# Molecular epidemiology of *Neisseria gonorrhoeae* using multi-antigen sequence typing and pulse-field gel electrophoresis in highly endemic Western Australian populations

**DOI:** 10.1186/s12879-015-0988-7

**Published:** 2015-07-15

**Authors:** Lyn C. O’Reilly, Namraj Goire, Rachel E. Fisk, David J. Speers

**Affiliations:** Department of Microbiology, PathWest Laboratory Medicine WA, Queen Elizabeth II Medical Centre, Hospital Avenue, Nedlands, 6009 WA Australia; School of Pathology and Laboratory Medicine, University of Western Australia, Crawley, 6009 WA Australia; School of Medicine and Pharmacology, University of Western Australia, Crawley, 6009 WA Australia

**Keywords:** *Neisseria gonorrhoea*, NG-MAST, PFGE, Typing, Epidemiology, *tbpB*, Western Australia

## Abstract

**Background:**

The remote and indigenous populations of Western Australia (WA) have one of the highest notification rates of gonorrhoea in the world. Despite this, the low rate of antimicrobial resistance in *Neisseria gonorrhoeae* from these regions permits the use of amoxycillin as empirical therapy. We describe the first molecular epidemiological study of gonococci isolated from this population using two different typing platforms.

**Methods:**

Pulse-field gel electrophoresis (PFGE), *Neisseria gonorrhoeae* multi-antigen sequence typing (NG-MAST) and antimicrobial susceptibility tests were performed on 128 consecutive *N. gonorrhoeae* isolates cultured between January 2011 and December 2013. To highlight clusters isolates were evaluated based on their *tbpB* sequence types.

**Results:**

No predominant NG-MAST or PFGE types were found. A total of 67 distinct PFGE pulsotypes were identified amongst the 128 isolates in this study with 20 PFGE pulsotypes representing 78 isolates. A total of 59 NG-MAST sequence types were found, represented by 45 *porB* alleles and 28 *tbpB* alleles with 13 *tbpB* genomogroups from 45 NG-MAST sequence types. *TbpB *genomogroup 29, represented by 45 isolates, was by far the most common genomogroup overall.

**Conclusions:**

Results from this study suggest that gonococcal epidemiology in WA is quite different between remote regions and major population centres and, in some cases, geographically restricted. It is likely that isolates originating from endemic regions of WA mostly represent independent, small sexual networks with an infrequent interchange between other communities and regions. Given the high rate of antimicrobial resistance elsewhere in Australia, ongoing surveillance is essential to ensure the enduring efficacy of amoxycillin empiric use in the remote regions of WA.

## Background

*Neisseria gonorrhoeae*, the etiological agent of the sexually transmitted disease gonorrhoea, has emerged as a challenging pathogen due to its propensity to acquire resistance to a wide range of antimicrobial agents. With the world-wide spread of antimicrobial resistant strains, epidemiological surveillance has acquired new importance for the control of gonorrhoea [1]. The remote and indigenous populations of Western Australia (WA), Australia’s largest state by area, have one of the highest notification rates of gonorrhoea in the world yet the circulating gonococci in these populations harbour very low levels of penicillin resistance [2]. As a result, amoxycillin combined with probenecid remains successful for the empiric management of gonorrhoeae acquired locally in these remote regions [3]. Moreover, to ensure the ongoing success of this treatment strategy surveillance for strains associated with antimicrobial resistance is required. This is especially so given the ongoing risk of resistant strain incursions from the more heavily populated metropolitan areas of WA and from the influx of inter-state and overseas workers for resource-mining related activities in these regions.

Most bacterial genotyping methods such as multi-locus sequence typing and pulse field-gel electrophoresis (PFGE) require live cultures. However, much of the gonococcal disease notification from remote region WA is based on molecular detection due to the difficulties in obtaining viable gonococci from remote regions. In this study *N. gonorrhoeae* multi-antigen sequence typing (NG-MAST) was compared to PFGE as NG-MAST has been successfully implemented on non-cultured clinical specimens [4]. Both methods have been used to identify transmission clusters to aid medico-legal investigations, but there is a lack of data comparing the performance of these methods for gonococcal typing [5–11]. In addition, *tbpB*-sequence based analysis was also performed as recent studies suggest that of the two loci used in NG-MAST study, *tbpB* exhibits lower variability and therefore can show distribution patterns of isolates. We examined the utility of *tbpB* sequence-based classification in determining disease transmission networks in these endemic populations [12, 13]. By employing PFGE, NG-MAST and *tbpB*-sequence based analysis, this study aimed to ascertain the molecular epidemiology of circulating gonococci from remote regional WA over the 3-year period from 2011 to 2013.

## Methods

### Study setting

**Fig. 1:**
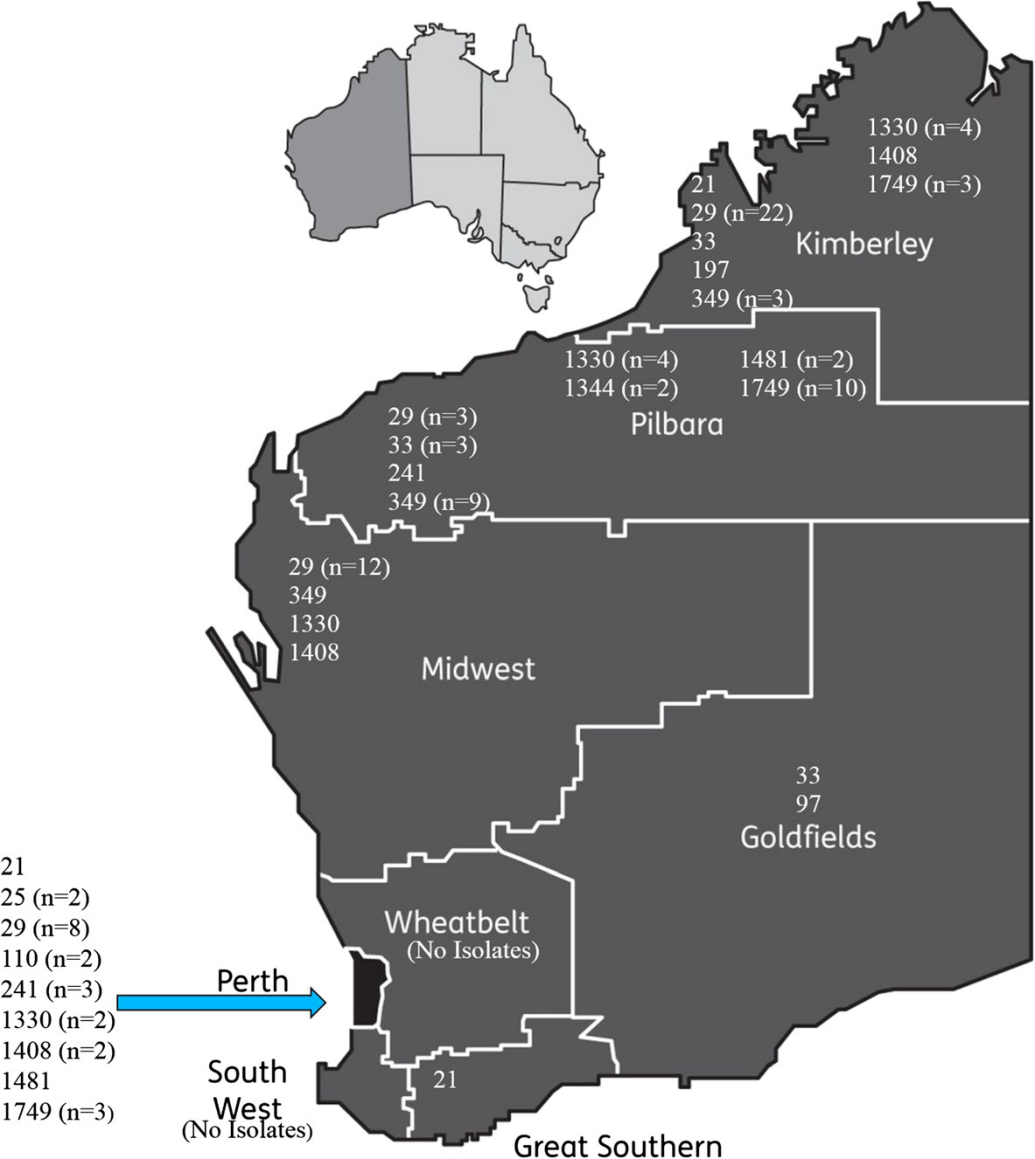
Distribution of *tbpB* groups across regions of Western Australia

PathWest Laboratory Medicine WA provides diagnostic and referral testing services to the majority of rural and remote WA populations. This includes the Pilbara and Kimberley regions which have some of the highest notification rates of gonorrhoea in the world [3]. Consecutive *N. gonorrhoeae* isolates cultured between January 2011 and December 2013 (*n* = 128) from both metropolitan (*n* = 30) and remote (*n* = 94) WA were included. The remote regions of WA represented in this study were the Goldfields (*n* = 4), Kimberley (*n* = 37), Mid-West (*n* = 17) and Pilbara (*n* = 36) (Fig. 1). A further four isolates were cultured from patients residing outside of WA. The vast majority of isolates (*n* = 115) were of genital origin with the remainder cultured from rectal swabs (*n* = 6), urine (*n* = 2), throat swabs (*n* = 2), blood culture (*n* = 1), knee aspirate (*n* = 1), and pelvic wash fluid (*n* = 1). The number of isolates cultured in 2011 (*n* = 36) and 2012 (*n* = 38) were comparable whereas there was an increase in isolate number in 2013 (*n* = 54). The patient population comprised of 86 males and 42 females.

### Bacterial identification

All *N. gonorrhoeae* isolates were cultured using standard microbiology laboratory protocols. The identification of bacterial colonies resembling *N. gonorrhoeae* was performed using at least two of the following identification platforms, Vitek2 NHI cards (Biomerieux), Phadebact gonococcal agglutination tests and Matrix-assisted laser desorption-ionisation test-of-flight (Bruker Deltonics, Bremen, Germany) following manufacturers’ instructions. The identity of each isolate was confirmed using a previously described *porA* gene specific *N. gonorrhoeae* real time PCR assay [14]. Antimicrobial susceptibility testing was performed on all isolates using the Etest method for penicillin and ceftriaxone following Central Laboratory Standards Institute guidelines and beta-lactamase activity was assayed using nitrocefin discs (Oxoid, Australia).

### NG-MAST

NG-MAST was performed as previously described [4] with modification. Briefly, each reaction mix consisted of 12 μL of PCR mix containing 0.2 mM of each forward and reverse primer (*porB*-Forward- CAAGAAGACCTCGGCAA and *porB*-Reverse- CCGACAACCACTTGGT for *porB* reaction and *tbpB*-Forward- CGTTGTCGGCAGCGCGAAAAC and *tbpB*-Reverse- TTCATCGGTGCGCTCGCCTTG for *tbpB* reaction), 0.5 units of DNA Polymerase (Applied Biosystems, USA), 2 mM Magnesium chloride (Invitrogen, USA), 1 unit of 10x PCR buffer (Applied Biosystems, USA), 0.01 % Bovine Serum Albumin (Sigma Aldrich, USA), 0.2 μM dNTP pool (Fisher Biotec, Australia), and 1 μM of 5-Carboxy-X-rhodamine succinimidyl ester (ROX) SYTO9 dye (Thermofisher Scientific, Australia). DNA lysate prepared by heat-denaturing gonococcal colonies (heating to 100 °C for 15 min) with 8 μL added to the PCR mix for a final reaction volume of 20 μL. Thermocycling was performed on a RotorGene 6000 real-time PCR instrument (Corbett Life Science, Australia) with the following cycling conditions; 95 °C for 15 min, followed by 45 cycles of 95 °C for 30 s, 60 °C for 30 s, and 72 °C for 1 min. PCR product formation was indicated by the presence of real-time PCR amplification curves. The amplified *porB* and *tbpB* sequences were then submitted for DNA sequencing using the ABI Prism BigDye Terminator v3.1 system (Applied Biosystems, Foster City, CA) according to the manufacturer’s instructions and the sequence data analysed using an ABI Prism 3130XL 16-channel Genetic Analyzer. The *porB* and *tbpB* sequences were trimmed to 490 bp and 390 bp respectively starting at particular sites of sequence conservation (TTGAA at the pre-loop 3 to loop 6 for *porB* and CGTCTGAA for *tbpB*) and then submitted to the NG-MAST website (http://www.ng-mast.net/). For all isolates returning previously unidentified (new) NG-MAST sequence types, the respective *porB* and *tbpB* sequences were submitted for addition to the NG-MAST database and assignment of a new sequence type (ST).

### PFGE typing

Samples were prepared using the standard PFGE method (PulseNet USA protocol, Centers for Disease Control and Prevention, Atlanta, Georgia. USA. 2004). Digestion with *Nhel* (Promega, Madison, USA) was performed overnight at 37 °C in 300 μL of fresh buffer containing 30 units of restriction endonuclease. The digested plugs were sealed into slots in 1 % agarose gel (Seakem Gold Agarose, Lonza, Rockland USA) and subjected to electrophoresis in a contour-clamped homogeneous electric field apparatus (BioRad, Hercules, California, USA). The electrophoresis was performed with pulse times ramping from 2.2 to 35 s for 18.3 h. Gels were stained with ethidium bromide. The *Nhel* enzyme produced approximately 15 to 20 distinct DNA fragments. Strains were considered identical if no fragment differences occurred. *Salmonella enteritidis ser Braenderup* H9812 was used as the reference ladder for normalisation. The results were analyzed using the BioNumerics software package version 6.6 (AppliedMaths, Kortrijk, Belgium) and the dendrograms were calculated using the unweighted-pair group method using the Dice coefficient. Pulsotypes (PT) were assigned numbers consecutively based on differences of more than one band in pulse-field patterns upon visual inspection.

### Statistical analysis

Diversity of both STs and PT were calculated using Simpsons’ index of diversity with an online calculator (http://darwin.phyloviz.net/ComparingPartitions/index.php?link=Home) as per a previously published method [15]. Discriminatory indices for PFGE and NGMAST were determined at 0.97 and 92.5 respectively.

### Ethics approval

No human material and only bacterial isolates originating from clinical specimens were used in this study. The use of human data in this study did not require an approval from the ethics committee of our organisation (Western Australian Health Department and the Queen Elizabeth II Medical Centre, Perth) as only subject data already obtained for diagnostic testing were used, and all data were de-identified.

## Results

### Bacterial identification and susceptibility tests

All isolates were confirmed as *N. gonorrhoeae* by both phenotypic methods and PCR. Reduced susceptibility or resistance to ceftriaxone was not identified. A total of 17 isolates (13 %) were resistant to penicillin, with 13 identified as penicillinase-producers and 4 exhibiting chromosomally mediated resistance. As expected, penicillin resistance was identified more often in metropolitan isolates (23 %) compared to remote region isolates (8 %).

### NG-MAST

The distribution of NG-MAST types across WA and over the study period was heterogeneous and lacked a common predominant ST. A total of 59 STs from the 128 isolates was found, represented by 45 *porB* alleles and 28 *tbpB* alleles. There were 15 STs shared by more than one isolate (*n* = 85, 66 %) while the remainder of STs were associated with single isolate each (Table 1). These 15 STs and the number of isolates assigned to them were as follows- ST 758 (*n* = 25), ST 4186 (*n* = 3), ST 5268 (*n* = 2), ST 7126 (*n* = 12), ST 7206 (*n* = 3), ST 7707 (*n* = 2), ST 7803 (*n* = 2), ST 8022 (*n* = 3), ST 8063 (*n* = 4), ST 9716 (*n* = 15), ST 10080 (*n* = 4), ST 10103 (*n* = 3), ST 10115 (*n* = 3), ST 10116 (*n* = 2) and ST 10117 (*n* = 2). ST 758 was the most numerous in the Perth (20 %), Mid-West (41 %) and Kimberley (29 %) regions with only one additional isolate belonging to this ST identified from the Pilbara region. The most common STs in Pilbara region were ST 7926 (25 %) and ST 9716 (25 %). There was minimal commonality of NG-MAST types between the Pilbara region and the neighbouring regions, apart from ST 9716 which was the second most common (8 %) ST in the Kimberley region.

**Table 1:** Distribution of NGMAST types, *tbpB* groups and their associated pulsotypes by year

Serial Number	Number of Isolates/year			Associated NGMAST types (n)^1^	*tbpB* Group (n)	Associated pulsotypes (n)^1^
	2011	2012	2013			
1	1	1	1	10111, 10115, 10118	Group 21 (3)	5, 12, 23
2	0	0	2	5268 (2)	Group 25 (2)	53 (2)
3	15	13	17	758 (25), 1498, 3042, 4822, 5961, 8063 (4) 7206 (3), 7353, 7707 (2), 10102, 10107, 10115 (2), 10123, 10124	Group 29 (45)	1, 2(2), 3(2), 5(3), 8(4), 12 (10), 13, 15 (3), 13, 16, 17, 19, 20, 22, 24, 34(2), 42, 49, 54, 65, 66, 68, 73(2), 76, 77
4	0	1	4	21, 5267, 8842, 5785, 10108	Group 33 (5)	29, 30, 46, 51, 73
5	0	0	2	3431, 5531	Group 110 (2)	13, 61
6	0	1	1	1214, 5368	Group 197 (2)	27, 63
7	0	2	2	4168 (3), 10121	Group 241 (4)	28(2), 47, 58
8	7	5	2	7126 (12), 7803 (2)	Group 349 (14)	4(6), 10(2), 21, 39(3), 50, 73
9	7	2	2	10080(4), 10103 (3), 10104, 10110, 10122, 10126	Group 1330 (11)	5(2), 12, 13, 14, 18, 73(5)
10	0	1	1	6943, 10112	Group 1344 (2)	26, 33
11	0	3	1	10116 (2), 10117 (2)	Group 1408 (4)	25, 48, 72(2)
12	1	0	2	8022 (3)	Group 1481 (3)	5(2), 8
13	2	3	11	9716 (15), 10113	Group 1749 (16)	8, 9(5), 12, 43, 44, 60, 71(5), 74
Total	33	32	48			

^1^number listed in brackets when associated isolates >1

### Classification based on *tbpB *sequences

There were 14 *porB* and 13 *tbpB* alleles that were associated with more than one isolate, accounting for 98 (76 %) and 113 (88 %) of all isolates respectively. The 13 *tbpB*-groups (Table 1) represented 45 of the STs. *TbpB*-group 29, represented by 45 isolates, was by far the most common genomogroup overall, being the most common group in the Metropolitan (*n* = 8), Kimberley (*n* = 22) and Mid-West (*n* = 12) regions (Fig. 1). In contrast, *tbpB*-groups 349 (*n* = 9) and 1749 (*n* = 10) were the most common in the Pilbara with only 3 *tbpB*-group 29 isolates from this region.

### PFGE typing

**Fig. 2:**
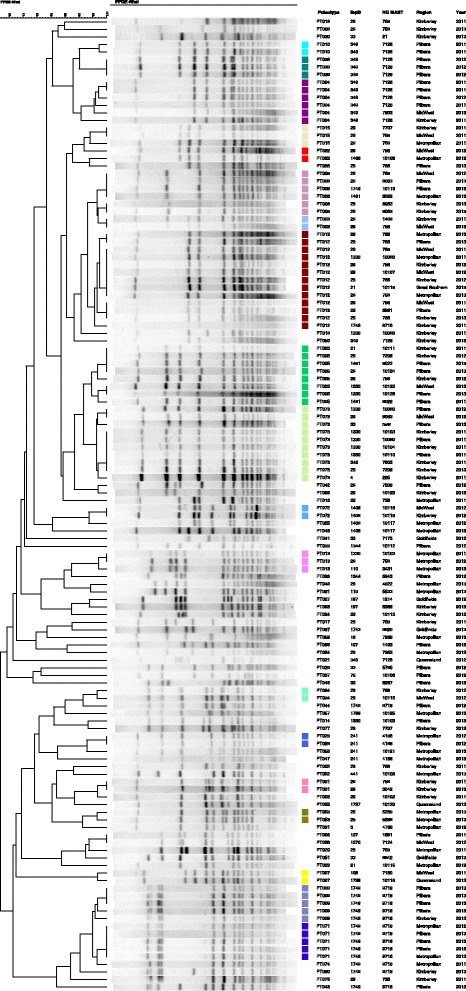
PFGE dendrogram of 128 *N. gonorrhoeae* isolates with associated *tbpB* and NG-MAST results and the region and year of their isolation. Dendogram is organised by pulsotypes with coloured squares highlighting groups of indistinguishable pulsotypes

A total of 67 distinct PFGE PT were identified amongst the 128 isolates in this study (Fig. 2). Twenty PT were identified in 78 isolates (60 %) in total. Similar to the NG-MAST results, no single band pattern predominated in any region. Six PT (4, 5, 8, 9, 12, 71 and 73) were each associated with more than five isolates, accounting for 41 % of all isolates. The distribution of PT across the regions of WA was very diverse with the predominant (more than four isolates) PT varying between the regions (Kimberley, PT5, PT73; Mid-West, PT12; Pilbara PT4, PT9). There was no predominant PT in the Goldfields or Metropolitan region. There was no correlation between PT and either STs or *tbpB* groups observed (Table 1) with the commonest *tbpB* group (*tbpB*-29) distributed across 25 different PT. Likewise, several PT were distributed among multiple *tbpB* groups.

## Discussion

We have described the first molecular epidemiological study of *N. gonorrhoeae* isolates from WA encompassing regions with some of the highest disease notification rates of gonorrhoea in the world. This study also represents the largest collection of *N. gonorrhoeae* isolates studied using the NG-MAST and PFGE methods in parallel.

The great diversity observed amongst the NG-MAST types in this study is most likely due to the hyper-variable nature of the target loci, especially the *porB* locus, evolving under selection pressure over years. Therefore we classified our isolates based on the relatively more conserved *tbpB* gene as this was more likely to demonstrate linkages over time [13]. Although NG-MAST still remains crucial for international comparison and tracking of strains of epidemiological importance, utility of single locus based typing for identifying closely-related clusters over a short period of time has been highlighted in studies from Canada and Germany [12, 13]. Of further interest was the gradual emergence of *tbpB*-group 1749 in this study, which was represented by 6 different PT whereas *tbpB* group 39 maintained relative stability in number over this three year period (Table 1).

Transmission of sexually transmitted disease like gonorrhoea is driven by a ‘core group’ of high risk individuals with high rates of infection and frequent partner change. A second ‘bridging group’, comprised of a population with lower risks, acts as the link between the general population and the core group [13, 16]. Identifying ‘core-group’ clusters is therefore very important in gonococcal disease transmission studies and is of particular relevance in remote communities where close-knit sexual networks are likely to exist [17]. The *tbpB*-group based classification identified the presence of four large clusters- namely *tbpB*-groups 29, 349, 1330 and 1749 which accounted for 67 % percent of all isolates in the study (Table 1). *TbpB*-group 29, the most numerous genomogroup in WA, could represent such a cluster in the Kimberley region where 11 out of 22 *tbpB*-group 29 isolates from this region originated from the same local precinct. Similarly, 8 out of the 12 *tbpB*-group 29 isolates from the Mid-West region were from two neighbouring precincts. In the Pilbara region, *tbpB*-groups 349 and 1749 together represented 19 isolates, 16 of which could be traced back to just two precincts. Of further interest, *tbpB*-group 29 was infrequently found amongst isolates from the Pilbara region (*n* = 3) despite its predominance in the surrounding Kimberley and Mid-West regions. Based on this, NGMAST has the potential to provide highly useful epidemiological information in our population, especially if applied on non-cultured clinical specimens which comprise the majority of notifications in WA.

Surprisingly, there was a major discordance between the PFGE and NG-MAST profiles of the isolates which remained unresolved despite reclassification into *tbpB*-groups. The constantly evolving NG-MAST loci are known to undergo changes to their *porB* and *tbpB* alleles over relatively short periods of time which would explain their distribution over a wide range of PT. Although PFGE exhibited higher discriminatory power compared to NG-MAST, based on this study, use of both methods is likely to yield more definitive results in medico-legal investigations [7, 8]. Further comparisons of the performance of these two methods over a larger population would possibly yield more conclusive results.

## Conclusion

The results from this study are most consistent with the hypothesis that the majority of the isolates originated from independent, small sexual networks with an infrequent interchange between other communities and regions. In terms of the *N. gonorrhoeae* genomogroups, it appears that some genomogroups were maintained in high transmission cycles, some were disappearing whereas others were establishing efficient transmission following recent introduction. No association could be made between antimicrobial susceptibility patterns and the ST, *tbpB*-group or PT.

A limitation of this study was that the cultured isolates included in this study comprise only a proportion of total gonorrhoea notifications in WA since most notifications in WA are based on molecular laboratory confirmation only. According to the Australian Gonococcal Surveillance Program (AGSP) the total number of notifications of gonorrhoeae in WA in the year 2012 was 548, thus the 38 isolates from 2012 in this study represented approximately 7 % [2]. Despite this, our study was able to show the diversity of *N. gonorrhoeae* strains in WA and suggest that the epidemiology of the various stains is quite different and, in some cases, geographically restricted. The application of NG-MAST or *tbpB* allele analysis to urogenital specimens uncultured but with detectable *N. gonorrhoea* DNA is worthy of further study as this would provide a more reliable picture of the prevalence and epidemiology of WA *N. gonorrhoeae* strains.

The low rates of antimicrobial resistance amongst the gonococci from remote and Aboriginal communities in WA has been attributed to both empirical use of dual amoxicillin/probenecid and azithromycin therapy as well as the social and geographical isolation from the mainstream urban populations [3, 18]. Incursion of strains harbouring antimicrobial resistance markers would therefore pose a serious public health challenge in this population. We did not find STs associated with international spread of multi-drug resistance phenotypes, such as ST 1407 or ST 225, in our remote populations although these strains have been described from the Eastern States of Australia [19, 20]. Therefore, given the history of spread of gonococcal antimicrobial resistance, a heightened vigil supported by a robust surveillance system is essential to ensure first line agents such as amoxycillin remain effective for our population.
